# Impact of Hospital Nurses’ Perception on Clinical Alarms and Patient Safety Culture on Alarm Management Practice

**DOI:** 10.3390/ijerph18084018

**Published:** 2021-04-12

**Authors:** Soo-Joung Lee, Yun-Mi Lee, Eun Ji Seo, Youn-Jung Son

**Affiliations:** 1 Division of Nursing, Inje University Haeundae Paik-Hospital, Busan 48108, Korea; Soo-joung@naver.com; 2 Institute of Health Science, College of Nursing, Inje University, Busan 47392, Korea; lym312@inje.ac.kr; 3 Research Institute of Nursing Science, College of Nursing, Ajou University, Suwon 16499, Korea; silbia98@ajou.ac.kr; 4 Red Cross College of Nursing, Chung-Ang University, Seoul 06974, Korea

**Keywords:** clinical alarms, nurses, perception, patient safety, nursing care, hospitals

## Abstract

This study aimed to identify the impact of nurses’ perception of clinical alarms and patient safety culture on alarm management. Additionally, we aimed to describe the importance of clinical alarm issues. The data were collected from 21 August to 10 September 2020. The study participants were 116 nurses working in a tertiary acute care hospital in Korea. The self-report questionnaire included general characteristics, clinical alarm issues, nurses’ alarm perception, patient safety culture, and alarm management practice. The mean age of nurses was 28.04 ± 4.06 years, with 5.71 ± 4.35 years of total clinical experience. For the importance of alarm issues, frequent false alarms leading to reduced attention or response was the most important issue. Hierarchical linear regression analysis revealed that a higher level of nurses’ perceived patient safety culture was the strongest predictor of better alarm management practice (*p* < 0.001), followed by their perception of clinical alarms (*p* = 0.034). In addition, female nurses (*p* = 0.004), charge nurses (*p* = 0.013), and nurses who work less than 40 h per week (*p* = 0.008) were more likely to work better in alarm management practice. Future studies are needed to develop standardized alarm management guidelines by improving nurses’ positive perceptions of clinical alarms and patient safety culture.

## 1. Introduction

The high acuity and complexity of patients who are admitted to tertiary care hospitals have increased significantly over time [1,2]. Improving early recognition and prompt interventions is therefore pivotal to the provision of quality care to a deteriorating patient before his condition becomes life-threatening [3,4]. To keep patients safe, equipment-related clinical alarms in hospitals are used to detect changes in patient condition and to monitor the proper functioning of equipment [5]. Clinical alarms in a hospital setting include physiological monitors, pulse oximetry, ventilators, and infusion pumps, which are primarily placed at patients’ bedsides to help provide them with optimal nursing care [6,7].

For healthcare professionals, responding to clinical alarms is driven by complex cognitive processes that make up the surveillance of the patient’s condition [8,9]. However, failure to respond to clinical alarms directly affects patients’ safety and satisfaction and can lead to a wide range of problems such as falls, pain, and heart attacks [10,11]. In addition, a high volume of clinical alarm signals that are false or insignificant can result in medical staff taking inappropriate actions such as ignoring the alarm signals [12,13]. Excessive clinical alarms can lead to alarm fatigue, a condition wherein healthcare professionals become desensitized by a high frequency of false alarms and alarms’ sounds, resulting in negative patient outcomes [14,15]. Considering that nurses are key healthcare professionals at patients’ bedsides, nurses are most exposed to numerous clinical alarms (the majority of which are false or nonactionable) [16,17,18]. Developing an alarm management protocol, building a multidisciplinary team for collaborating with the medical device manufacturer, and adjusting alarms, including alarm settings, limits, and delays, are known strategies to avoid alarm fatigue [19,20,21]. Optimal clinical alarm management may be required for reducing patient harm and preventing nurses’ alarm fatigue [19,20].

To date, regardless of the resources available to individual institutions, alarm safety or effective clinical alarm management is a key priority [7,19]. However, many studies have focused on clinical alarm-related issues in intensive care units [21,22,23,24]. Alarm management is a complex issue that can reduce alarm fatigue and has a significant impact on patient safety in clinical settings [19,21]. Nurses have a primary responsibility in the recognition and prevention of patient deterioration [24,25,26]. In this regard, nurses should be able to respond timely to clinical alarms to maintain awareness of the potential risk of inappropriate alarm settings [27,28]. Effective clinical alarm management is a challenging area for hospital nurses and requires understanding and application of clinical alarm safety practice [7,13]. For this reason, previous studies emphasized that staff education is vital to increasing the awareness of alarm issues, as well as informing staff of alarm management practices [16,17]. Thus, nurses’ perceptions of clinical alarms that may influence alarm recognition and response are crucial for alarm management [29]. However, nurses’ perspectives on alarm management practices have not adequately addressed all nursing staff, including general wards and intensive care units (ICUs).

Patient safety is defined as the absence of preventable harm to patients and preventing unnecessary harm by healthcare professionals [30,31]. In this regard, the development of a patient safety culture has also been considered as an effective strategy to improve patient safety [30]. Patient safety culture is defined as the shared values, beliefs, and behavioral norms related to patient safety among members of an organization, unit, or team [31]. In particular, safety culture behaviors such as risk management, including reporting errors, are essential to establish a positive patient safety culture [4,31]. Therefore, more research is needed to identify which patient safety culture, as shared behavioral norms, is significantly associated with safety competency regarding alarms among hospital nurses.

In Korea, the Patient Safety Act was announced in July 2016 [32], a foundation for systematically managing patient safety problems at the national level [32]. The Act established a nationwide voluntary error-reporting system; however, the focus remains still on cause analysis. Furthermore, little research has been conducted on topics related to clinical alarms. The main aim of this study was to identify the impact of nurses’ perception of clinical alarms and patient safety culture on alarm management practice. In addition, we aimed to investigate the importance of clinical alarm issues among hospital nurses.

## 2. Methods

### 2.1. Study Design and Setting

This study has a cross-sectional design. This study was conducted in a tertiary hospital located in Busan, Korea from 21 August 2020 to 10 September 2020.

### 2.2. Participants and Data Collection

The sample was drawn from the population of registered nurses who regularly worked in a department using medical equipment such as infusion or syringe pumps and bedside patient monitors to set a clinical alarm. Nurses were excluded if they were working in the emergency room. Patients’ length of stay in an emergency room is shorter, and experiences with medical devices are quite different from that in the medical or surgical general wards and critical care units. In addition, pediatric care units were also excluded due to their having different medical devices compared to adult care units.

The sample size was computed using the G*power v. 3.14 software [33]. With a statistical significance of 0.05, a power of 0.8, and a medium effect size of 0.15 for multiple linear regression, the minimum estimated sample size was 103 participants. Based on this calculation, and to allow for a 20% dropout rate, a total of 124 structured questionnaires containing recruiting notices and informed consent forms were distributed to four nursing departments (two general wards and two ICUs) in a single hospital. Nurses were invited to participate voluntarily. After completing the questionnaires, the participants returned them in a sealed envelope to a primary investigator (return rate 97.6%). Among the 121 returned questionnaires, five individuals who answered the questionnaire were excluded from the analysis because of incomplete responses. Finally, 116 participants were included in the data analysis.

### 2.3. Instruments

#### 2.3.1. General Characteristics

General characteristics included age, gender, education, job title, type of unit, total clinical experience, clinical experience at the current unit, and weekly working hours.

#### 2.3.2. Clinical Alarm-Related Important Issues

The importance of clinical alarm issues was measured using a scale developed by Healthcare Technology Foundation [34]. The Korean version of the scale [11] was validated by the expert group (one bilingual translator, two nursing supervisors, and two nursing professors) and composed of questions that nurses recognized as important with regard to the problems that hinder alarm management of medical equipment (e.g., the excessive number of false alarms). Nurses were asked to rate nine issues that inhibit effective alarm management using a nine-point scale from 1 (most important) to 9 (least important). The average ranking was measured by summing up the rankings assigned to each of the nine questions and measuring the average value, and then assigning them from first to ninth in the order of lowest average.

#### 2.3.3. Alarm Perception

Nurses’ perception of clinical alarms was measured using the National Clinical Alarms Survey of the Healthcare Technology Foundation [35]. We used the Korean version validated by Cho et al. [11]. Perceptions about clinical alarms signaled from all monitoring devices were probed. For example, “Good understanding of what each alarm means” and “Alarms in my department contribute to my stress level”. Each item was rated on a 5-point Likert scale, from 1 (strongly disagree) to 5 (strongly agree). The higher the score, the more negative the alarm perception. In this study, Cronbach’s α value was 0.76.

#### 2.3.4. Perceived Patient Safety Culture

Nurses’ perceived patient safety culture was measured using the survey instrument to assess Korean patient safety culture for hospitals developed by Lee [36]. This tool was validated with content validity and construct validity and reviewed by an expert panel comprised 10 patient safety experts in academic and clinical settings [36]. This 35-item tool measures leadership, teamwork, patient safety knowledge/attitude, patient safety policy/procedure, non-punishment environment, patient safety improvement system, and patient safety priority. Each item was rated on a 5-point Likert scale, from 1 (strongly disagree) to 5 (strongly agree). The scores ranged from 35 to 175, with a higher score indicating a higher level of patient safety culture. The Cronbach’s α of the original study was 0.94. The Cronbach’s α was 0.89 in the present study.

#### 2.3.5. Alarm Management Practices

Alarm management was measured using Alarm Management, originally developed by Han [37] based on international guidelines [38,39]. Namely, the questions related to nurses’ alarm management practice were based on professional recommendations and current care standards. This 22-item tool measures the alarm management practice for the electrocardiogram (EKG) monitor (9 items), pulse oximetry (4 items), mechanical ventilator (3 items), and other devices’ clinical alarms (6 items). Each item was rated on a 4-point Likert scale, from 1 (not at all) to 4 (always). Scores ranged from 22 to 88, with a higher score indicating higher alarm management. The Cronbach’s α was 0.75 in the present study.

### 2.4. Ethical Considerations

This study was approved by the institutional review board of Inje University in Korea (Approval no: 2020-04-032-001). Potential study participants were informed that participation in the study was voluntary and that the data would be used only for the present study. Study participants read and signed a statement of informed consent. Completed questionnaires were identified through the study identification number and placed in an anonymous envelope before distribution to ensure the anonymity and confidentiality of the participants.

### 2.5. Data Analysis

All statistical analyses were performed using the Statistical Package for Social Sciences software 25.0 (IBM Corp., Armonk, NY, USA). Participants’ general characteristics, clinical alarm-related important issues, alarm perception, patient safety culture, and alarm management practice were presented with frequencies, percentage, mean, and standard deviation. Cronbach’s α was used to assess the internal consistency reliability of the measurement scales. The minimum criterion for acceptable reliability is an α of at least 0.70 [40]. Alarm management practice by nurses’ general characteristics was analyzed using an independent t-test and analysis of variance (ANOVA) with the Scheffé post-hoc test. Relationships among alarm perception, patient safety culture, and alarm management practice were examined using Pearson’s correlation coefficients. The factors influencing alarm management practice were analyzed through hierarchical multiple regression.

## 3. Results

### 3.1. General Characteristics of Nurses

The mean age of the nurses was 28.04 ± 4.06 years old; 109 nurses (94.0%) were female. Eighty-eight nurses (75.9%) had a bachelor’s degree. A total of 101 nurses (87.1%) were staff nurses and 47 nurses (59.4%) worked in the medical and surgical intensive care unit. The mean years of total clinical experience was 5.71 ± 4.35 years, the mean years of clinical experience at current department was 4.28 ± 3.37 years, and the mean hours of recent weekly working hours was 36.67 ± 9.65 h (Table 1).

### 3.2. Clinical Alarm-Related Important Issue

The most important issue that nurses recognized as difficult in alarm management was the “Frequent false alarms, which lead to reduced attention or response to alarms when they occur”, followed by “Overreliance on alarms to call attention to patient problems”. Conversely, the least important issue was “Difficulty in hearing alarms when they occur” (Table 2).

### 3.3. Descriptive and Correlation Among Alarm Perception, Patient Safety Culture, and Alarm Management Practice

The mean score of alarm perception and patient safety culture among nurses was 2.87 (standard deviation [SD] = 0.42) and 3.61 (0.31), respectively. The mean score of the alarm management practice was 2.77 (0.30). Among the subcategories of alarm management practice, other clinical alarms had the highest mean score (3.24), followed by pulse oximetry (2.94), mechanical ventilator (2.87), and EKG monitor (2.34) (Table 3). Alarm management practice was significantly negatively correlated with alarm perception (r = −0.20, *p* = 0.033) and positively correlated with patient safety culture (r = 0.29, *p* = 0.001) (Table 3).

### 3.4. Differences in Alarm Management Practice According to Nurses’ General Characteristic

The alarm management practice showed significant differences according to gender (t = −2.05, *p* = 0.043), job title (t = −2.37, *p* = 0.020), type of unit (F = 2.82, *p* = 0.042), and recent weekly working hours (F = 4.01, *p* = 0.021) (Table 4).

### 3.5. Impact of Alarm Perception and Patient Safety Culture on Alarm Management Practice among Nurses

In the first model, gender, job title, type of unit, and weekly working hours, which were significant differences with regard to alarm management practice (*p* < 0.05) were entered. Next, in the second model, alarm perception and patient safety culture were entered into a hierarchical linear regression in order to identify the contribution of the two main study variables. In Model 1, women (*p* = 0.010), charge nurses (*p* = 0.007), and over 41 h of weekly working hours (*p* = 0.012) were significantly associated with nurses’ alarm management practice. They explained 15% of the variance in nurses’ alarm management. In Model 2, alarm perception (*p* = 0.034) and patient safety culture (*p* < 0.001) were significantly associated with nurses’ alarm management practice after controlling for the nurses’ general characteristics. This explained the additional 13% of the variance in nurses’ alarm management practices (Table 5).

## 4. Discussion

Our main finding showed that nurses’ positive perceptions of clinical alarms and higher patient safety culture levels were associated with better alarm management practice among hospital nurses after controlling for general characteristics. Nurses are key healthcare professionals managing multiple monitoring devices [24,25,26]. Effective alarm management can improve patient safety and the quality of care in hospital settings [10,11]. Thus, nurses should understand and apply clinical alarm management practices.

Positive alarm perception means that nurses recognize that the alarms accurately inform the patients’ condition or help them perform effective nursing activities. It also means that nurses know the meaning of the alarms and are not afraid of the alarm being activated [2,13]. This can be easily linked to alarm management practices. This is in line with previous studies [24,26]. Therefore, education on the situation in which alarms of various equipment may occur is required, first, to understand the meaning of the alarms and cope with it. When education and training clarify regarding what should be done and what false alarms are in each alarm situation, nurses are allowed to perform the alarm management activities naturally for patients. In fact, adjusting settings based on patient condition was an effective strategy to minimize alarm volume and to respond in a timely manner [13,22,28].

In the present study, patient safety culture had the greatest impact on alarm management practice. In a well-developed patient safety culture, nurses can clearly recognize that all nursing activities are directly related to patient safety and take action when necessary [30,31]. Besides, nurses can face positive pressure from other healthcare providers and departments that shape their safety culture [4]. Therefore, alarm management activity, one of the nursing behaviors, can be performed more in a positive patient safety culture. This is in line with a previous study that found that hospitals should ensure an ethically sensitive climate because the nurses’ perception was relevant to the hospitals that they belonged to [41]. A culture in which nurses can easily communicate about and support each other in alarm management would make it convenient to come to the most acceptable way of using alarms. Therefore, there is a need for a system that analyzes and improves alarm management as one of the indicators of patient safety culture. In addition, since a direct supervisor is one of the factors that can greatly affect the patient safety culture [42], it is necessary to organize a program for nurses to participate in patient safety-related education with the head of their unit and to discuss alarm management practices as safety activities.

In addition, women, charge nurses, and nurses who work less than 40 h a week on average were associated with better practices in alarm management. While other variables in this study were similarly distributed, the participants were predominantly women. Charge nurses are registered nurses responsible for the nursing unit’s operation over a specific period [43]. In this regard, charge nurses may be more sensitive to alarms from the patient because of their role that expects them to perform a multitude of tasks while serving as important unit leaders [43]. It can also be understood that the difference in career did not significantly affect alarm management practice. However, the results of this study were different from those of a previous study that showed that the types of departments such as wards and intensive care units were related to alarm management practice [27]. It is possible that this is because vital equipment, including portable EKGs, is also used in the ward since the severity of hospitalized patients is high due to the aging population and their multi-morbidities. However, alarm management practice did not have a high score, with an average of 2.77 out of the 4 points. In particular, since the score of the EKG management is the lowest at 2.34 points, it is necessary to strengthen education on important equipment, including the EKG monitoring device.

The nurses’ perceptions of important alarm issues in this study similar to previous studies were frequent false alarms, overreliance on alarms, and inadequate staff to respond to alarms [11,27]. False alarms and overreliance can be issues related to patient conditions and the appropriate alarm settings with regard to their conditions. Therefore, to resolve the alarm issue, it is necessary to set appropriate alarms according to the patient’s condition and to have the ability to understand these alarms. The manufacturers of devices also need to improve alarm-related issues by facilitating an open platform to discuss the nurses’ needs for alarm settings. Meanwhile, overreliance on medical devices and inadequate staff may be issues related to the appropriateness of nursing staffing. Maintaining continuously adequate staffing according to the severity of the patient’s condition is necessary for proper alarm management. In fact, proper staffing and education were the measures most suggested by nurses to improve alarm perception and response [13]. Nurses need to integrate clinical support with an educational framework for physiologic monitoring and alarm safety [44]. Hence, the hospital management also needs to provide a safe environment with critical provisions that cater to the needs of nurses.

There are several limitations to this study. First, nurses’ patient safety culture and alarm management practices were assessed using a self-reported measure that can be influenced by memory recall. Moreover, they might not reflect actual patient safety culture and alarm management practices. Second, the reliability and validity of the three main variables were assessed using Cronbach’s alpha. The lack of other reliability and validity estimates is a limitation of this study. Third, this study included a small sample size, using a single Korean university hospital with convenience sampling. In addition, most of the respondents were female, young, and held a bachelor’s degree. For these reasons, future research needs to confirm the hypotheses across a wider sample, including a prospective multicenter study design. Although this study has several limitations, this is the first study to elucidate the associations between nurses’ alarm perception and patient safety culture with alarm management practices among Korean hospital nurses.

## 5. Conclusions

For effective clinical alarm management, a multidimensional complex process is required. Our findings highlight that fostering nurses’ positive perceptions of clinical alarms and patient safety culture may improve the practice of alarm management at the individual level. It is recommended to develop a standardized tool that assesses nurses’ perception of clinical alarms and alarm management guidelines. At the organizational level, individualized and unit-specific training should be developed. Hospital administrators could also pay more attention to frontline nurses’ needs, including that of other healthcare professionals, taking into account the critical context for better patient safety and work efficiency.

## Figures and Tables

**Table 1:** Demographic and work-related characteristics of nurses (N = 116).

Characteristics	Categories	*n* (%)	M ± SD
Age (year)	22–24	21 (18.1)	28.04 ± 4.06
	25–29	61 (52.6)	
	30–34	27 (23.3)	
	≥35	7 (6.0)	
Gender	Women	109 (94.0)	
	Men	7 (6.0)	
Education	Diploma	25 (21.6)	
	Bachelor Degree	88 (75.9)	
	≥Master Degree	3 (2.6)	
Job title	Staff Nurse	101 (87.1)	
	Charge Nurse	15 (12.9)	
Type of unit	Medical General Ward	21 (18.1)	
	Surgical General Ward	26 (22.4)	
	Medical ICU	36 (31.0)	
	Surgical ICU	33 (28.4)	
Total clinical experience (year)	<3	36 (31.0)	5.71 ± 4.35
	3~4	28 (24.1)	
	5~9	30 (25.9)	
	≥10	22 (19.0)	
Clinical experience at current department (year)	<3	51 (44.0)	4.28 ± 3.37
	3~4	28 (24.1)	
	5~9	25 (21.6)	
	≥10	12 (10.3)	
Weekly working hours	≤32	33 (28.4)	36.67 ± 9.65
	33~40	67 (57.8)	
	≥41	16 (13.8)	

Note: Staff nurse: a registered nurse employed by a hospital that provides direct patient care.

**Table 2:** Clinical alarm-related important issues (N = 116).

Item Statement	Item Response Mean	Mean Ranking ^†^
Frequent false alarms, which lead to reduced attention or response to alarms when they occur	2.81	1
Overreliance on alarms to call attention to patient problems	4.64	2
Inadequate staff to respond to alarms as they occur	4.69	3
Difficulty in understanding the priority of an alarm	4.85	4
Lack of training on alarm systems	5.18	5
Difficulty in setting alarms properly	5.22	6
Noise competition from nonclinical alarms and pages	5.45	7
Difficulty in identifying the source of an alarm	5.68	8
Difficulty in hearing alarms when they occur	6.48	9

ICU = intensive care unit; ^†^ = Item response means were ranked from 1 (most important) to 9 (least important).

**Table 3:** Description and correlation among alarm perception, patient safety culture, and alarm management practice (N = 116).

Variables (Items)	Min.–Max. (Range)	M ± SD	Alarm Perception	Patient Safety Culture
			R (*p*)	R (*p*)
Alarm perception (14 items)	1.44–3.78 (1–5)	2.87 ± 0.42	1	
Patient safety culture (35 items)	2.80–4.94 (1–5)	3.61 ± 0.31	0.12 (0.208)	1
Alarm management practice (22 items)	1.68–3.55 (1–4)	2.77 ± 0.30	−0.20 (0.033)	0.29 (0.001)
EKG monitor (9 items)	1.67–2.89 (1–4)	2.34 ± 0.26		
Pulse oximetry (4 items)	1.75–4.00 (1–4)	2.94 ± 0.46		
Mechanical ventilator (3 items)	1.00–4.00 (1–4)	2.87 ± 0.68		
Other clinical alarms (6 items)	1.50–4.00 (1–4)	3.24 ± 0.51		

Note: The scores of alarm perception and patient safety culture range from 1 (strongly disagree) to 5 (strongly agree). The alarm management practice was a score between 1 (not at all) and 4 (always).

**Table 4:** Differences in alarm management practice according to general characteristics (N = 116).

Characteristics	Categories	M ± SD	t or F (*p*)	Scheffé Test
Age (year)	22–24	2.77 ± 0.21	2.41 (0.070)	
	25–29	2.73 ± 0.31		
	30–34	2.77 ± 0.30		
	≥35	3.05 ± 0.27		
Gender	Female	2.75 ± 0.30	−2.05 (0.043)	
	Male	2.99 ± 0.15		
Education	Diploma	2.79 ± 0.26	1.96 (0.146)	
	Bachelor’s Degree	2.77 ± 0.31		
	≥Master’s Degree	2.44 ± 0.23		
Job title	Staff Nurse	2.74 ± 0.30	−2.37 (0.020)	
	Charge Nurse	2.93 ± 0.26		
Type of unit	Medical general ward ^a^	2.81 ± 0.29	2.82 (0.042)	c < d
	Surgical general ward ^b^	2.74 ± 0.30		
	Medical ICU ^c^	2.67 ± 0.25		
	Surgical ICU ^d^	2.87 ± 0.32		
Total clinical experience (year)	<3	2.77 ± 0.22	0.26 (0.854)	
	3~4	2.74 ± 0.32		
	5~9	2.75 ± 0.36		
	≥10	2.81 ± 0.29		
Clinical experience at current department (year)	<3	2.79 ± 0.26	0.66 (0.579)	
	3–4	2.71 ± 0.32		
	5–9	2.80 ± 0.35		
	≥10	2.72 ± 0.27		
Weekly working hours	≤32 ^a^	2.87 ± 0.35	4.01 (0.021)	a > c
	33–40 ^b^	2.75 ± 0.25		
	≥41 ^c^	2.63 ± 0.30		

Note: Staff nurse: a registered nurse employed by a hospital that provides direct patient care. ICU = intensive care unit.

**Table 5:** Hierarchical linear regression analysis for nurses’ alarm management practice (N = 116).

Predictors	Model 1		Model 2		*R*^2^ Change	F (*p*)	*R*^2^ (Adjusted *R*^2^)
	Standardized Beta	t(*p*)	Standardized Beta	t(*p*)			
Gender, women	0.24	2.64 (0.010)	0.25	2.93 (0.004)	0.15	4.74 (0.001)	0.15 (0.12)
Job title, charge nurse	0.24	2.73 (0.007)	0.21	2.54 (0.013)			
Type of unit, ICU	−0.09	−1.06 (0.292)	−0.15	−1.78 (0.077)			
Weekly working hours, ≥41	−0.23	−2.26 (0.012)	−0.22	−2.72 (0.008)			
Alarm perception	-	-	−0.18	−2.15 (0.034)	0.13	7.01 (<0.001)	0.28 (0.24)
Patient safety culture	-	-	0.34	4.16 (<0.001)			

## Data Availability

Due to privacy and ethical concerns, neither the data nor the source of the data can be made available.
